# Data on the effect of climate change-related variables on the abundance of antibiotic resistance genes in a manure-amended soil

**DOI:** 10.1016/j.dib.2025.112358

**Published:** 2025-12-07

**Authors:** Fernando Ruiz-Torrubia, Carlos Garbisu, Lur Epelde

**Affiliations:** Department of Conservation of Natural Resources, NEIKER – Basque Institute for Agricultural Research and Development, Basque Research and Technology Alliance (BRTA), Parque Científico y Tecnológico de Bizkaia, P812, 48160 Derio, Spain

**Keywords:** Agroecosystems, Antibiotic resistant bacteria, Antimicrobial resistance, Moisture, Resistome, Soil microbes, Temperature

## Abstract

This article presents a dataset of antibiotic resistance gene abundances obtained when exposing soil, previously amended with oxytetracycline-spiked cow manure, to different temperatures and moisture contents as two highly relevant climate change-related variables. The absolute abundances of six antibiotic resistance genes (ARGs) and two mobile genetic element (MGE)-linked genes were determined by droplet-digital PCR. Data on soil microbial biomass carbon, the total abundance of the 16S rRNA gene, and basal respiration are also included to show the effect of the climate change-related variables on the biomass and activity of soil microbial communities. The dataset presented in this article contains raw observations (including the soil´s physicochemical characterization), as well as analysis-derived data, on the effects of climate change-related variables on the risk of antibiotic resistance occurrence and spread in soils amended with animal manure, a topic of the utmost importance given the potential links between the environmental resistome and the human resistome. The data provided in this article are of much interest to researchers dealing with the potential impact of agricultural practices (i.e., organic fertilization) on antibiotic resistance under the current scenario of climate change.

## Graphical abstract

**Figure:**
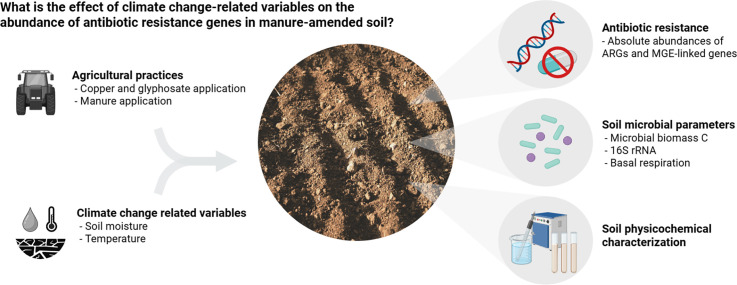

Specifications Table

**Table:** 

Subject	Earth & Environmental Sciences
Specific subject area	Impact of climate change on antibiotic resistance in manure-amended soils.
Type of data	Raw data, tables, graphs, and figures.
Data collection	Soil physicochemical characterization was performed according to standard methods. The abundances of ARGs and MGE-linked genes were measured by droplet-digital PCR. Soil microbial biomass carbon was determined by the fumigation-extraction method. The total abundance of the 16S rRNA gene was determined by real-time qPCR. Soil basal respiration was determined by quantifying CO_2_ evolution according to ISO 16,072.
Data source location	The soil was collected from the upper 20 cm of a grassland located in Derio (northern Spain). All determinations were performed at NEIKER´s facilities (Derio).
Data accessibility	Repository name: https://zenodo.org/ Data identification number: 10.5281/zenodo.15754293 Direct URL to data: https://zenodo.org/records/15,754,293
Related research article	None.

## 1 Value of the Data

• The data provided in this article are of much interest to researchers dealing with the impact of agricultural practices (i.e., manure application as organic fertilizer) on antibiotic resistance under the current scenario of climate change. These data are of great value for the development and refining of predictive models to assess the potential impact of climate change on antimicrobial resistance, incorporating previously overlooked factors such as the role of the environmental resistome. Additionally, these data could inform the development of policies aimed at addressing the global threat of antimicrobial resistance within the One Health framework.
• The presented data address separately the effects of two highly relevant climate change-related variables: temperature and moisture. By isolating these factors, researchers can better understand the specific mechanisms through which climate change-related variables may alter the abundance and spread of ARGs in organically-amended agricultural soils.
• The data include the effects of the climate change-related variables on soil microbial biomass, the total abundance of the 16S rRNA gene, and basal respiration, contributing to our understanding of the responses of manure-amended agricultural soils in terms of microbial biomass and activity to climate change-induced disturbances.
• The data are of much interest to researchers working on organic farming and, specifically, those dealing with the unintended adverse effects of organic fertilization such as, for instance, its potential contribution to the antibiotic resistance problem.

## 2 Background

Since the adoption of the One Health framework to tackle the antibiotic resistance (AR) problem, the role of the environmental component of AR has gained much attention. Nowadays, the environment is considered an important reservoir of AR determinants, and then, the abundance and diversity of such determinants in the environment, together with the risk of their transfer to human bacterial pathogens, must be evaluated [1].

Agroecosystems are being thoroughly studied regarding their contribution to the AR problem. Agricultural practices, such as the application of manure-based fertilizers, have been shown to contribute to the emergence and spread of ARB and ARGs in soils and crops [2], as manure itself can (i) be a hotspot for the presence of ARGs and ARB; and (ii) contain antibiotic residues, as well as their transformation products, originating from veterinary treatments. Manure treatments such as aging, composting, and the addition of amendments can modify the load of ARGs, ARB, and antibiotic residues [3]. In this respect, organic farming can unintendedly open a route for AR transference to human bacterial pathogens through the consumption of agricultural crops.

On the other hand, certain alterations in climate change-associated variables, such as increases in global temperature, have been associated with an increased prevalence of AR determinants [[4], [5], [6]]. This association has been attributed to factors such as altered rates of bacterial reproduction, horizontal gene transfer, changes in bacterial transmission through biological vectors, and behavioral or social shifts that can promote the spread of AR [5]. However, not many studies have examined how climate change-related variables influence the environmental resistome. Li et al. and Hacopian et al. reported that elevated soil temperatures can increase both the abundance and expression of certain ARGs in forest soils [7,8]. In contrast, the effects of other climatic factors, such as soil moisture, remain unclear: some studies suggest that soil moisture drives ARG abundance, whereas others find no significant relationship [[9], [10], [11]]. Understanding how climate-associated variables shape the emergence and dissemination of AR in agroecosystems is crucial for elucidating the role of the environmental resistome on these processes, as well as for informing policies aimed at mitigating the spread of resistance. The presented data address separately the effects of temperature and moisture to better understand the mechanisms through which climate change-related variables may alter the abundance of ARGs in organically-amended soils.

## 3 Data Description

This article presents a dataset of antibiotic resistance gene abundances obtained when exposing soil, previously amended with oxytetracycline-spiked cow manure, to different temperatures and moisture contents as two highly relevant climate change-related variables. The metadata of the dataset are available in the file Metadata within the uploaded dataset. Initially, the soil was subjected to physicochemical characterization, including soil nutrients (nitrogen, Olsen phosphorus, potassium, magnesium, and calcium), pH, cation exchange capacity, electrical conductivity, organic matter content, particle size distribution, and texture (Table 1). The manure used in this study had a total carbon content of 10.44 % and a total nitrogen content of 0.54 %. This is consistent with previous studies that report that a C/N ratio of 19 is common for cattle manures [12]. The application of organic amendments with C/N ratios moderately higher than the typical soil C/N ratio of 8–10 can enhance soil microbial growth and activity [13].

**Table 1:** Soil physicochemical characterization.

Parameter	
Soil organic matter (%)	5.03
Dry matter (%)	80.29
Total carbon (%)	7.20
Total nitrogen (%)	0.37
Ammonium (mg N—NH_4_^+^ kg^−1^)	4.72
Nitrate (mg N—NO_3_^−^ kg^−1^)	26.33
Carbonate (%)	30.27
C/N ratio	7.90
Olsen phosphorus (mg kg^−1^)	23.75
Potassium (mg kg^−1^)	171.35
Magnesium (mEq 100 g^−1^)	1.16
Calcium (mEq 100 g^−1^)	34.29
pH in water (1:2.5, w/v)	7.97
Effective cation exchange capacity (COHEX) (mEq 100 g^−1^)	24.64
Electrical conductivity in water (mS cm^−1^)	0.19
Active lime (%)	7.89
Clay (%)	25.70
Fine sand (%)	31.65
Coarse sand (%)	5.10
Silt (%)	37.56
Textural class	Loam

**Fig. 1:**
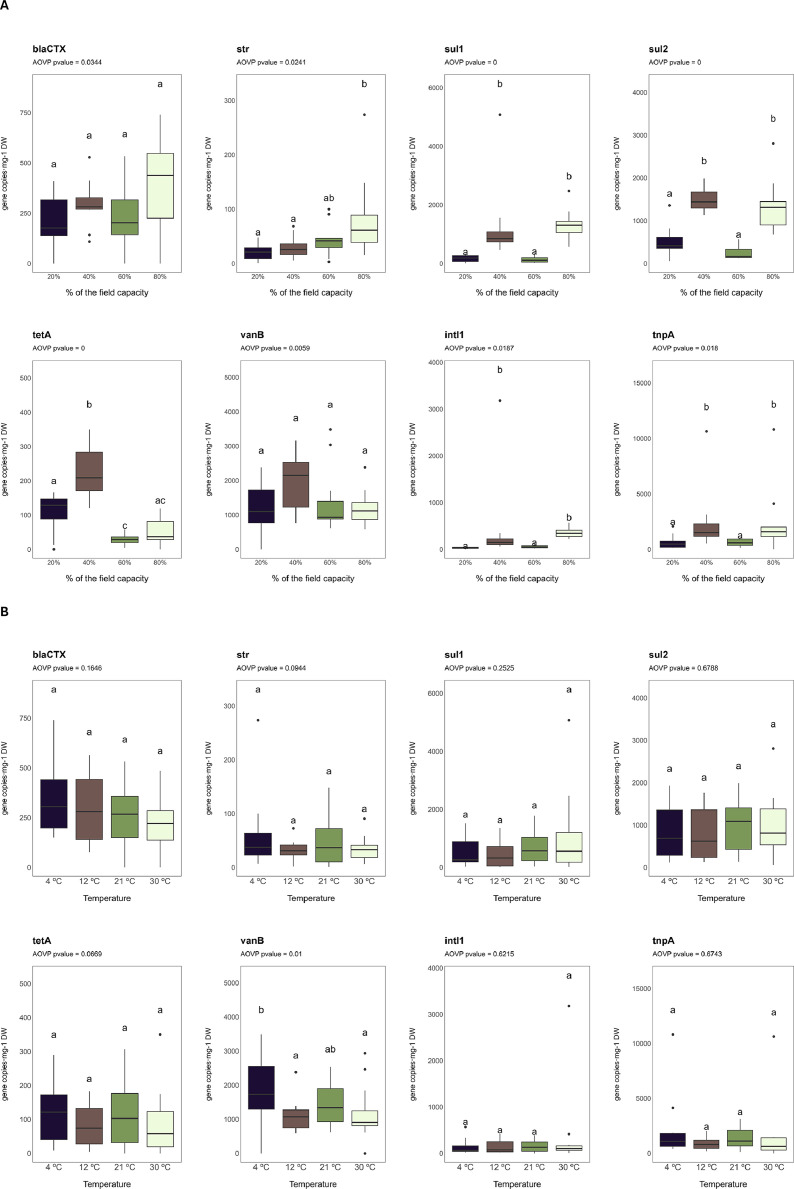
Absolute abundances of ARGs and MGE-linked genes in soil samples with distinct: (A) moisture levels; and (B) incubation temperatures. Different letters indicate statistically significant (*p* < 0.05) differences among treatments according to Dunn’s *post hoc* test.

The mean (*n* = 3) absolute abundances of the measured ARGs and MGE-linked genes are presented in Table 2, while raw measurements are available in the Data_ddPCR file within the uploaded dataset. Statistically (*p* < 0.05) significant differences between samples with distinct moisture levels or incubated at different temperatures were determined with a permutation ANOVA test and are shown in Fig. 1. The absolute abundances of the measured genes were found to be significantly different (*p* < 0.05) between samples with distinct moisture levels. The effect of moisture on these absolute abundances was generally non-linear (Fig. 1): *sul1, sul2, intl1*, and *tnpA* genes exhibited significantly higher absolute abundances at 40 % and 80 % field capacity (FC), compared to 20 % and 60 % FC, while the highest absolute abundance of *tetA* gene was observed at 40 % FC. This may be because the abundance of these genes depends on the growth and gene transfer conditions of their microbial hosts, which are diverse and may have different optimal requirements. In contrast, *str* gene exhibited its highest absolute abundance at 80 % FC, and the lowest at 20 % and 40 % FC, while *blaCTX* and *vanB* genes showed no statistically significant differences across moisture levels (Fig. 1). Regarding differences between samples incubated at different temperatures, only the absolute abundance of *vanB* varied significantly among treatments, which may be explained by the optimal growth and gene transfer conditions of the bacterial hosts carrying this gene. The highest absolute abundance of *vanB* was observed in samples incubated at 4 °C (Fig. 1).

**Table 2:** Absolute abundances of ARGs and MGE-linked genes measured by droplet-digital PCR in soil samples (no. copies mg^−1^ of dry soil). Mean (*n* = 3) ± standard deviation. Statistically significant differences were determined with a permutation ANOVA test and are represented as *(*p* < 0.1), **(*p* < 0.05), and ***(*p* < 0.01). FC: Field capacity.

Moisture	Temperature	*blaCTX*	*str*	*sul1*	*sul2*	*tetA*	*vanB*	*intI1*	*tnpA*
20 % of the FC	4 ºC	237 ± 150	13 ± 9	237 ± 38	454 ± 159	149 ± 22	1253 ± 1094	44 ± 15	598 ± 208
	12 ºC	191 ± 150	23 ± 20	73 ± 89	391 ± 159	98 ± 42	1335 ± 921	30 ± 9	181 ± 4
	21 ºC	147 ± 129	20 ± 25	252 ± 78	726 ± 548	135 ± 30	1465 ± 244	28 ± 25	1134 ± 823
	30 ºC	265 ± 110	26 ± 6	145 ± 170	494 ± 393	49 ± 74	576 ± 502	33 ± 33	645 ± 690
40 % of the FC	4 ºC	329 ± 41	42 ± 19	929 ± 319	1564 ± 324	255 ± 54	1782 ± 1206	99 ± 33	1487 ± 263
	12 ºC	259 ± 233	26 ± 7	692 ± 259	1449 ± 314	158 ± 26	1103 ± 316	161 ± 58	917 ± 547
	21 ºC	321 ± 79	34 ± 32	1085 ± 407	1588 ± 423	254 ± 46	2504 ± 47	239 ± 105	2658 ± 485
	30 ºC	274 ± 5	16 ± 16	2207 ± 2484	1404 ± 228	215 ± 120	2413 ± 552	1156 ± 1753	4299 ± 5503
60 % of the FC	4 ºC	313 ± 191	100 ± 0	98 ± 86	134 ± 12	37 ± 26	2742 ± 928	36 ± 23	636 ± 217
	12 ºC	334 ± 115	26 ± 21	82 ± 64	150 ± 27	25 ± 4	969 ± 297	34 ± 10	985 ± 76
	21 ºC	142 ± 168	27 ± 18	134 ± 117	273 ± 170	22 ± 15	818 ± 172	80 ± 45	515 ± 422
	30 ºC	149 ± 22	59 ± 28	223 ± 121	435 ± 139	30 ± 17	944 ± 105	88 ± 23	359 ± 119
80 % of the FC	4 ºC	532 ± 290	123 ± 131	1119 ± 562	1181 ± 604	60 ± 51	1804 ± 542	389 ± 161	5607 ± 4652
	12 ºC	417 ± 158	53 ± 17	1080 ± 438	1145 ± 408	47 ± 47	1019 ± 374	365 ± 51	1503 ± 695
	21 ºC	460 ± 103	113 ± 33	1296 ± 418	1285 ± 257	46 ± 53	1167 ± 318	294 ± 88	1414 ± 535
	30 ºC	174 ± 270	36 ± 22	1655 ± 712	1661 ± 1031	57 ± 30	769 ± 145	417 ± 0	926 ± 795
**Treatment or interaction**		***blaCTX***	***str***	***sul1***	***sul2***	***tetA***	***vanB***	***intI1***	***tnpA***
Temperature							**		
Moisture		*	**	***	***	***	***	***	**
Temperature:moisture							**		*

**Fig. 2:**
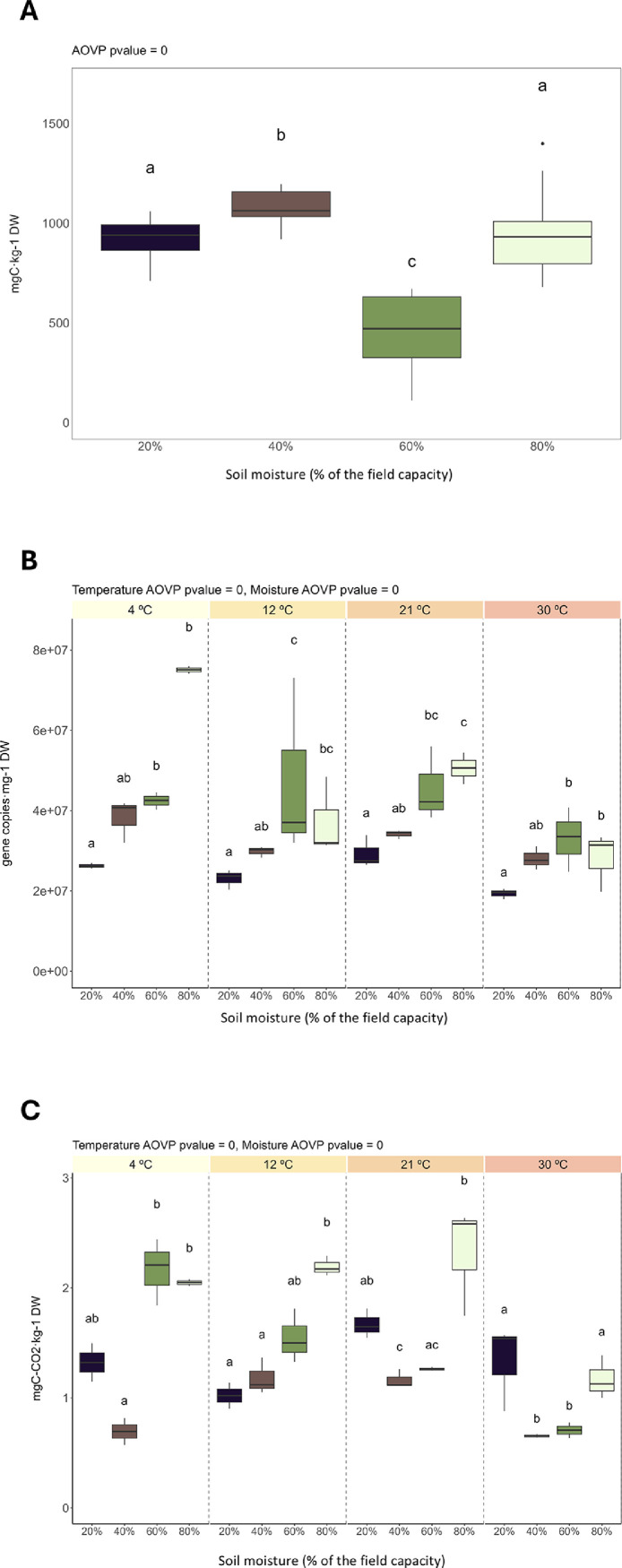
Soil microbial parameters: (A) microbial biomass carbon (mg C kg^−1^ dry soil) and (B) total abundance of the 16S rRNA gene (gene copies g^−1^ dry soil) as indicators of soil microbial biomass; and (C) basal respiration (mg C—CO_2_ kg^−1^ dry soil) as indicator of soil microbial activity. Different letters indicate statistically significant (*p* < 0.05) differences among treatments according to Dunn’s *post hoc* test.

The mean (*n* = 3) values of the measured soil microbial parameters (i.e., microbial biomass carbon, total abundance of the 16S rRNA gene, basal respiration) are presented in Table 3, while the raw measurements are available in the Data_Microbial Parameters file within the uploaded dataset. Statistically significant (*p* < 0.05) differences between samples with distinct moisture levels or incubated at different temperatures were determined with a permutation ANOVA test and are shown in Fig. 2. Microbial biomass carbon was significantly different (*p* < 0.05) between samples with distinct moisture levels (though this moisture effect was non-linear), while no differences were observed between samples incubated at different temperatures. Regarding the 16S rRNA gene, its total abundance was significantly different (*p* < 0.05) between samples with distinct moisture levels and incubated at distinct temperatures. Soil basal respiration also showed significant differences depending on moisture level and incubation temperature, with a significant interaction between these two variables.

**Table 3:** Soil microbial parameters. Microbial biomass carbon (mg C kg^−1^ dry soil); Basal respiration (mg C—CO_2_ kg^−1^ dry soil); 16S rRNA: gene copies of the 16S rRNA gene (copies g^−1^ dry soil). Mean (*n* = 3) ± standard deviation. Statistically significant differences were determined with a permutation ANOVA test and are represented as *(*p* < 0.1), **(*p* < 0.05), and ***(*p* < 0.01). FC: Field capacity.

Moisture	Temperature	Microbial biomass C	16S rRNA	Basal respiration
20 % of the FC	4 ºC	999 ± 44	26,266,295 ± 687,461	1.32 ± 0.25
	12 ºC	864 ± 1	23,044,415 ± 2448,417	1.02 ± 0.17
	21 ºC	990 ± 65	29,291,197 ± 3999,312	1.67 ± 0.13
	30 ºC	827 ± 120	19,314,161 ± 1278,698	1.33 ± 0.39
40 % of the FC	4 ºC	1149 ± 59	38,161,864 ± 5401,646	0.69 ± 0.17
	12 ºC	1076 ± 66	29,812,663 ± 1360,384	1.18 ± 0.16
	21 ºC	1065 ± 94	34,126,021 ± 1062,698	1.16 ± 0.08
	30 ºC	1014 ± 85	28,030,654 ± 2918,649	0.65 ± 0.01
60 % of the FC	4 ºC	389 ± 39	42,466,835 ± 2170,440	2.16 ± 0.30
	12 ºC	460 ± 220	47,367,455 ± 22,423,236	1.55 ± 0.24
	21 ºC	476 ± 316	45,512,849 ± 9317,672	1.26 ± 0.01
	30 ºC	465 ± 254	33,042,124 ± 8010,095	0.71 ± 0.10
80 % of the FC	4 ºC	1167 ± 327	75,067,266 ± 1331,840	2.05 ± 0.04
	12 ºC	878 ± 179	37,276,532 ± 9641,891	2.19 ± 0.09
	21 ºC	814 ± 86	50,570,111 ± 3914,939	2.32 ± 0.50
	30 ºC	1012 ± 239	28,169,487 ± 7340,754	1.17 ± 0.20
**Treatment or interaction**		**Microbial biomass C**	**16S rRNA**	**Basal respiration**
Temperature			***	***
Moisture		***	***	***
Temperature:moisture			***	***

## 4 Experimental Design, Materials and Methods

### 4.1 Experimental design

In this experiment, four moisture levels (i.e., 20, 40, 60, and 80 % of the soil´s field capacity − FC) and four incubation temperatures (i.e., 4, 12, 21, and 30 °C) were tested. The combination of moisture and incubation temperatures resulted in 16 different treatments. Two months before the start of the experiment, an aged cow manure was spiked with oxytetracycline to reach a final concentration of 2000 mg kg^−1^. At the beginning of the experiment, the soil was dried until it reached 20 % of its FC. At this point, copper (added as CuSO_4_) and glyphosate (added as Clinic®, Nufarm) were applied to the soil to simulate common agricultural practices, i.e., the use of Cu-based fungicides and the use of herbicides. First, a CuSO_4_ water solution was mixed with sand, then dried, and mixed with the soil to reach a final concentration of 100 mg Cu kg^−1^ soil. The glyphosate was diluted in distilled water and applied to the soil to give a final concentration of 20 mg kg^−1^ soil. Oxytetracycline-spiked manure was incorporated to the soil at the beginning of the assay at a rate of 204 t ha^−1^, or 14.5 % w/w. Subsequently, 300 *g* of soil were put in each pot, with three replicates per treatment. The pots were incubated in the absence of light at four temperatures (i.e., 4, 12, 21, and 30 °C) and watered every 3 days throughout the experiment to maintain the specific moisture level of each treatment. After 11 weeks, soil samples were collected, sieved to < 2 mm, and kept at 4 °C for less than one month prior to analytical determinations.

### 4.2 Soil collection and characterization

Soil was collected from the upper 20 cm of a grassland located in Derio (northern Spain) and sieved to < 6 mm to ensure homogenization. A portion of this soil, designated for physicochemical characterization, was air-dried at 30 °C for 48 h, and further sieved to < 2 mm. Total carbon and nitrogen contents were determined by combustion with a TruSpec CHN Analyser (LECO Corporation, Michigan, USA) in accordance with Hidalgo et al. [14]. Soil pH and electrical conductivity were determined in water at 1:2.5 w/v and 1:5 w/v, respectively, following Hidalgo et al. [14]. Olsen phosphorus and particle size distribution were also determined following Hidalgo et al. [14]. The determination of soil organic matter and carbonate concentration was conducted in accordance with DIN 19,539 [15]. Soil extractable potassium, magnesium, and calcium were determined by Inductively Coupled Plasma Atomic Emission Spectrometry (ICP-AES) in accordance with standard methods [16]. Effective cation exchange capacity was determined according to ISO 23,470 [17]. The determination of nitrate concentration was conducted with a UV–VIS Spectrophotometer UV-1800 (Shimadzu), at a wavelength of 220 nm, following Cawse [18]. Lastly, the measurement of ammonium concentration was conducted in accordance with Nelson [19]. The aged cow manure was obtained from NEIKER facilities in Derio (northern Spain). The fraction designated for the determination of carbon and nitrogen contents (by combustion with a TruSpec CHN Analyser) was air-dried at 30 °C for 48 h, and then sieved to < 2 mm.

## 5 Determination of Soil Microbial Parameters

Soil microbial biomass carbon and the total abundance of the 16S rRNA gene were determined as indicators of soil microbial biomass [14]. Soil microbial biomass carbon was determined by the fumigation-extraction method. DNA from soil samples was extracted using the DNeasy PowerSoil Pro Kit (Qiagen, Carlsbad, CA, USA). Following extraction, DNA quantity and quality were determined using a NanoDrop™ One Spectrophotometer (Thermo Scientific, Wilmington, DE, USA). Extracted DNA samples were stored at −20 °C until use. The total abundance of the 16S rRNA gene was determined by real-time qPCR. The primers used for the amplification were Ba519f (CAGCMGCCGCGGTAANWC) and Ba907r (CCGTCAATTCMTTTRAGTT), with an annealing temperature of 50 °C and a resulting amplicon of 424 bp. Soil basal respiration, as indicator of soil microbial activity, was determined by measuring CO_2_ evolution in hermetic flasks incubated at 30 °C for 72 h.

### 5.1 Determination of the absolute abundance of ARGs and MGE-linked genes

Gene quantification by ddPCR was carried out according to Jauregi et al. [3]. First, DNA samples were digested with the *XbaI* restriction enzyme (TakaraBio, CA, USA) according to the manufacturer’s instructions. Then, 1 µl of the digested sample was mixed with 10.5 µl of sterile Milli-Q water, 12.5 µl of ddPCR EvaGreen Supermix (Bio-Rad), and 0.5 µl of each of forward or reverse primers (final concentration 10 nM each). Eight primer couples were used, six of them targeting ARGs (*blaCTX-M, str, sul1, sul2, tetA,* and *vanB*) and two targeting MGE-linked genes (*intl1*, and *tnpA*) (Table 4). Then, 21 µl of the PCR reaction mixture were withdrawn and dispensed into a DG8 Droplet Generation Cartridge (Bio-Rad). Droplet generation was performed according to the manufacturer’s instructions. The generated droplets were transferred to a 96-well plate, which was then sealed and introduced in the C1000 Touch Thermal Cycler for PCR amplification. The plate was then placed in a QX200 droplet reader for data acquisition. Data analysis was performed using the QuantaSoft software (v.1.4., Bio-Rad).

**Table 4:** Targeted genes measured by droplet-digital PCR and primer sets used.

Gene	Primer (5′→3′)	Sequence	Reference
*blaCTX-M*	Forward	GCGATAACGTGGCGATGAAT	[20]
	Reverse	GTCGAGACGGAACGTTTCGT	
*str*	Forward	AATGAGTTTTGGAGTGTCTCAACGTA	[20]
	Reverse	AATCAAAACCCCTATTAAAGCCAAT	
*sul1*	Forward	CCGTTGGCCTTCCTGTAAAG	[21]
	Reverse	TTGCCGATCGCGTGAAGT	
*sul2*	Forward	CGGCTGCGCTTCGATT	[22]
	Reverse	CGCGCGCAGAAAGGATT	
*tetA*	Forward	GCTACATCCTGCTTGCCTTC	[23]
	Reverse	CATAGATCGCCGTGAAGAGG	
*vanB*	Forward	TTGTCGGCGAAGTGGATCA	[24]
	Reverse	AGCCTTTTTCCGGCTCGTT	
*intI1*	Forward	GCCTTGATGTTACCCGAGAG	[25]
	Reverse	GATCGGTCGAATGCGTGT	
*tnpA*	Forward	CCGATCACGGAAAGCTCAAG	[26]
	Reverse	GGCTCGCATGACTTCGAATC	

## Limitations

This dataset presents the effect of two climate change-related variables on the absolute abundance of eight genes (6 ARGs, 2 MGE-linked genes) connected with the AR problem, but future studies must evaluate the effect of these variables on a much broader range of genes (e.g., the soil resistome) to draw more comprehensive and robust conclusions. Additionally, it should be noted that this study did not assess the effect of the experimental factors on antibiotic degradation dynamics. These dynamics affect the persistence of antibiotics in soils and may consequently influence the environmental resistome data. Besides, the risk that the environmental resistome poses to human health greatly depends on the actual transfer of ARGs from environmental bacteria to human bacterial pathogens. Then, more research must be directed to study the effects of climate change-related variables on the mechanisms that mediate ARG transfer between bacteria or between ecological spaces. This could be achieved by addressing horizontal gene transfer or measuring AR in different ecological spaces. Lastly, the effects shown in this dataset correspond to a microcosm experiment in which soil samples were exposed to the climate change-related variables for only 11 weeks. To draw robust conclusions, these data should be replicated in a field experiment following long-term exposure to the climate change-related variables.

## Ethics Statement

The authors confirm that they have complied with the ethical requirements for publication in Data in Brief, stating that the present work does not involve human participants, animal experimentation, or data obtained from social media platforms.

## CRediT Author Statement

**Fernando Ruiz-Torrubia:** Conceptualization, formal analysis, writing – original draft; **Carlos Garbisu:** Conceptualization, funding acquisition, writing – review and editing; **Lur Epelde:** Conceptualization, funding acquisition, writing – review and editing.

## Data Availability

ZenodoJune 27, 2025 (v1)DatasetEmbargoed Data on the effect of climate change-related variables on the abundance of antibiotic resistance genes in a manure-amended soil (Original data).

## References

[1] Larsson D.G.J., Flach C.F. (2022). Antibiotic resistance in the environment. Nat. Rev. Microbiol..

[2] Wang F., Fu Y.-H., Sheng H.-J., Topp E., Jiang X., Zhu Y.-G., Tiedje J.M. (2021). Antibiotic resistance in the soil ecosystem: a one health perspective. Curr. Opin. Env. Sci..

[3] Jauregi L., González A., Garbisu C., Epelde L. (2023). Organic amendment treatments for antimicrobial resistance and mobile element genes risk reduction in soil-crop systems. Sci. Rep..

[4] Li W., Liu C., Ho H.C., Shi L., Zeng Y., Yang X., Huang Q., Pei Y., Huang C., Yang L. (2023). Association between antibiotic resistance and increasing ambient temperature in China: an ecological study with nationwide panel data. Lancet Reg. Health West. Pac..

[5] MacFadden D.R., McGough S.F., Fisman D., Santillana M., Brownstein J.S. (2018). Antibiotic resistance increases with local temperature. Nat. Clim. Change.

[6] McGough S.F., MacFadden D.R., Hattab M.W., Mølbak K., Santillana M. (2020). Rates of increase of antibiotic resistance and ambient temperature in Europe: a cross-national analysis of 28 countries between 2000 and 2016. Eurosurveillance.

[7] Li Z., Sun A., Liu X., Chen Q.-L., Bi L., Ren P.-X., Shen J.-P., Jin S., He J.-Z., Hu H.-W., Yang Y. (2022). Climate warming increases the proportions of specific antibiotic resistance genes in natural soil ecosystems. J. Hazard. Mater..

[8] Hacopian M.T., Barrón-Sandoval A., Romero-Olivares A.L., Berlemont R., Treseder K.K. (2025). Warming is associated with more encoded antimicrobial resistance genes and transcriptions within five drug classes in soil bacteria: a case study and synthesis. Environ. Microbiol..

[9] Reichel R., Radl V., Rosendahl I., Albert A., Amelung W., Schloter M., Thiele-Bruhn S. (2014). Soil microbial community responses to antibiotic-contaminated manure under different soil moisture regimes. Appl. Microbiol. Biotechnol..

[10] Radl V., Kindler R., Welzl G., Albert A., Wilke B.-M., Amelung W., Schloter M. (2015). Drying and rewetting events change the response pattern of nitrifiers but not of denitrifiers to the application of manure containing antibiotic in soil. Appl. Soil Ecol..

[11] McKinney C.W., Dungan R.S. (2020). Influence of environmental conditions on extracellular and intracellular antibiotic resistance genes in manure-amended soil: a microcosm study. Soil Sci. Soc. Am. J..

[12] R. Rynk, M. van de Kamp, G.B. Willson, M.E. Singley, T.L. Richard, J.J. Kolega, F.R. Gouin, L. Laliberty, D. Kay, D.W. Murphy, H.A.J. Hoitink, W.F. Brinton, On-farm Composting Handbook (NRAES 54), Northeast Regional Agricultural Engineering Service, Ithaca, N.Y., 1992.

[13] Quemada M., Delgado A., Mateos L., Villalobos F.J., Villalobos F.J., Fereres E. (2024). Principles of Agronomy for Sustainable Agriculture.

[14] Hidalgo J., Artetxe U., Becerril J.M., Gómez-Sagasti M.T., Epelde L., Vilela J., Garbisu C. (2023). Biological remediation treatments improve the health of a mixed contaminated soil before significantly reducing contaminant levels. Environ. Sci. Pollut. Res. Int..

[15] DIN 19539:2016-12. Investigation of solids. Temperature-dependent differentiation of total carbon, (2016).

[16] Ministerio de Agricultura, Pesca y Alimentacion (MAPA), Métodos Oficiales de Análisis de Suelos y Aguas Para Riego, in: Métodos Oficiales de Análisis III, Ministerio de Agricultura, Pesca y Alimentacion, 1994.

[17] International Organization for Standardization, ISO 23470:2018. Determination of effective cation exchange capacity (CEC) and exchangeable cations using a hexamminecobalt trichloride solution, (2018).

[18] Cawse P.A. (1967). The determination of nitrate in soil solutions by ultraviolet spectrophotometry. Analyst.

[19] Nelson D.W. (1983). Determination of ammonium in KCl extracts of soils by the salicylate method. Commun. Soil Sci. Plant Anal..

[20] Stedtfeld R.D., Guo X., Stedtfeld T.M., Sheng H., Williams M.R., Hauschild K., Gunturu S., Tift L., Wang F., Howe A., Chai B., Yin D., Cole J.R., Tiedje J.M., Hashsham S.A. (2018). Primer set 2.0 for highly parallel qPCR array targeting antibiotic resistance genes and mobile genetic elements. FEMS Microbiol. Ecol..

[21] Heuer H., Smalla K. (2007). Manure and sulfadiazine synergistically increased bacterial antibiotic resistance in soil over at least two months. Environ. Microbiol..

[22] Heuer H., Focks A., Lamshöft M., Smalla K., Matthies M., Spiteller M. (2008). Fate of sulfadiazine administered to pigs and its quantitative effect on the dynamics of bacterial resistance genes in manure and manured soil. Soil Biol. Biochem..

[23] Ng L.-K., Martin I., Alfa M., Mulvey M. (2001). Multiplex PCR for the detection of tetracycline resistant genes. Mol.Cell. Probes.

[24] Wang F.-H., Qiao M., Su J.-Q., Chen Z., Zhou X., Zhu Y.-G. (2014). High throughput profiling of antibiotic resistance genes in urban park soils with reclaimed water irrigation. Environ. Sci. Technol..

[25] Barraud O., Baclet M.C., Denis F., Ploy M.C. (2010). Quantitative multiplex real-time PCR for detecting class 1, 2 and 3 integrons. J. Antimicrob. Chemother..

[26] Zhu Y.-G., Johnson T.A., Su J.-Q., Qiao M., Guo G.-X., Stedtfeld R.D., Hashsham S.A., Tiedje J.M. (2013). Diverse and abundant antibiotic resistance genes in Chinese swine farms. Proc. Natl. Acad. Sci. U.S.A..

